# Dried Bilberry (*Vaccinium myrtillus* L.) Alleviates the Inflammation and Adverse Metabolic Effects Caused by a High-Fat Diet in a Mouse Model of Obesity

**DOI:** 10.3390/ijms231911021

**Published:** 2022-09-20

**Authors:** Toini Pemmari, Mari Hämäläinen, Riitta Ryyti, Rainer Peltola, Eeva Moilanen

**Affiliations:** 1 The Immunopharmacology Research Group, Faculty of Medicine and Health Technology, Tampere University and Tampere University Hospital, 33014 Tampere, Finland; 2 Bioeconomy and Environment, Natural Resources Institute Finland, 96100 Rovaniemi, Finland

**Keywords:** bilberry, *Vaccinium myrtillus* L., high-fat diet, liver inflammation, non-alcoholic fatty liver disease, hyperglycemia, hypercholesterolemia

## Abstract

Obesity is an increasing problem worldwide. It is often associated with co-morbidities such as type II diabetes, atherosclerotic diseases, and non-alcoholic fatty liver disease. The risk of these diseases can be lowered by relieving the systemic low-grade inflammation associated with obesity, even without noticeable weight loss. Bilberry is an anthocyanin-rich wild berry with known antioxidant and anti-inflammatory properties. In the present study, a high-fat-diet-induced mouse model of obesity was used to investigate the effects of air-dried bilberry powder on weight gain, systemic inflammation, lipid and glucose metabolism, and changes in the gene expression in adipose and hepatic tissues. The bilberry supplementation was unable to modify the weight gain, but it prevented the increase in the hepatic injury marker ALT and many inflammatory factors like SAA, MCP1, and CXCL14 induced by the high-fat diet. The bilberry supplementation also partially prevented the increase in serum cholesterol, glucose, and insulin levels. In conclusion, the bilberry supplementation alleviated the systemic and hepatic inflammation and retarded the development of unwanted changes in the lipid and glucose metabolism induced by the high-fat diet. Thus, the bilberry supplementation seemed to support to retain a healthier metabolic phenotype during developing obesity, and that effect might have been contributed to by bilberry anthocyanins.

## 1. Introduction

The prevalence of obesity has increased remarkably during the last decades. Obesity is associated with severe co-morbidities such as atherosclerotic diseases, type II diabetes, and non-alcoholic fatty liver disease (NAFLD) [1,2,3,4,5,6]. Atherosclerotic diseases are associated with inflamed, dysfunctional adipose tissue, hypertriglyceridemia, and elevated cholesterol levels [1]. Type II diabetes and its defining feature, elevated blood glucose, are closely related to decreased insulin sensitivity, which desynchronizes the delicate cross-talk between the hepatic, adipose, and muscular tissues and may finally lead to the exhaustion of the pancreas and dependence on external insulin [2]. NAFLD is a deleterious condition that may lead to cirrhosis and/or cancer in the liver. The accumulation of lipids and nontriglyceride lipotoxicity injure hepatocytes and activate resident macrophages known as Kupffer cells. Kupffer cells then produce and secrete chemokines, cytokines, and other inflammatory factors that attract circulating inflammatory cells into the liver and promote inflammation [4,6]. Although obesity is a well-known risk factor of the diseases mentioned, there is emerging evidence of the concept of healthy obesity i.e., a situation where overweight seems to cause remarkably fewer metabolic adverse effects than expected [1]. The presence of systemic inflammation is one of the essential features of unhealthy obesity [1]. Thus, weight loss may not be the only treatment for the typical obesity-associated diseases, but shifting the metabolism towards the healthier phenotype could relieve human and financial burden caused by obesity and its co-morbidities.

Wild berries, particularly bilberry (*Vaccinium myrtillus* L.), contain anthocyanins and other polyphenols that have many beneficial effects, including anti-inflammatory and vasodilatory actions. Epidemiological studies have shown an inverse correlation between the intake of certain polyphenols and obesity-related diseases such as atherosclerotic diseases [7]. The major anthocyanins of bilberry have been studied in the context of inflammation as separate molecules and as anthocyanin combinations in vitro and in vivo [8]. Anthocyanins often potentiate the activity of each other when present simultaneously [9]. Extrapolating the effects of whole berries from the data achieved with single anthocyanins may thus be an underestimation. Additionally, wild berries have been an important part of the daily diet in many countries for decades [10].

The aim of the present study was to examine the effects of bilberry supplementation in the high-fat-diet model of obesity in mice, focusing on lipid and glucose metabolism, as well as inflammation. The results showed that bilberry alleviated inflammation, especially in the liver, and retarded the development of unwanted changes in the lipid and glucose metabolism.

## 2. Results

### 2.1. Body Weight and Food Intake

**Figure 1:**
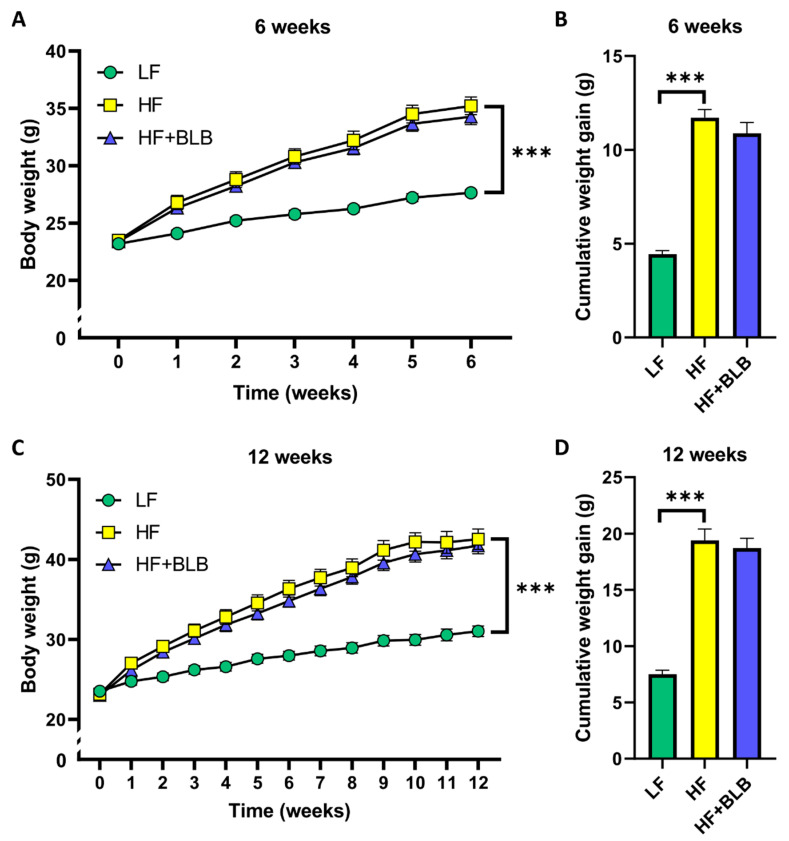
The body weight gain during the study. The mice received a low-fat diet (LF, 10% of energy from fat, circle), a high-fat diet (HF, 46% of energy from fat, square) or a bilberry-supplemented high-fat diet (HF+BLB, 20% air-dried bilberry powder, triangle) for six (**A**,**B**) or twelve (**C**,**D**) weeks. The weights were analyzed with repeated measures, such as a two-way (mixed model) ANOVA and a Bonferroni multiple comparisons test (A,C). The cumulative weight gain was analyzed with a one-way ANOVA and a Bonferroni multiple comparisons test (**B**,**D**). The statistically significant differences from the HF group are marked with *** *p* < 0.001. The results are expressed as grams; values represent mean ± SEM, n = 12 mice in each group.

The mice were fed a low-fat diet (LF diet, 10% energy from fat), a high-fat diet (HF diet, 46% of energy from fat) or a bilberry-supplemented high-fat diet (HF+BLB diet, 20% of weight air-dried bilberry powder) for six or twelve weeks. The weight of the mice receiving the HF diet increased remarkably compared to the LF group during the six-week treatment (*p* < 0.001). The bilberry supplementation of the HF diet made no difference to the weekly or cumulative weight gain compared to the HF group (Figure 1A,B). The disparities between the LF and HF groups continued to increase in the longer, twelve-week treatment (*p* < 0.001), and the bilberry supplementation had no effect on body weight (Figure 1C,D).

**Figure 2:**
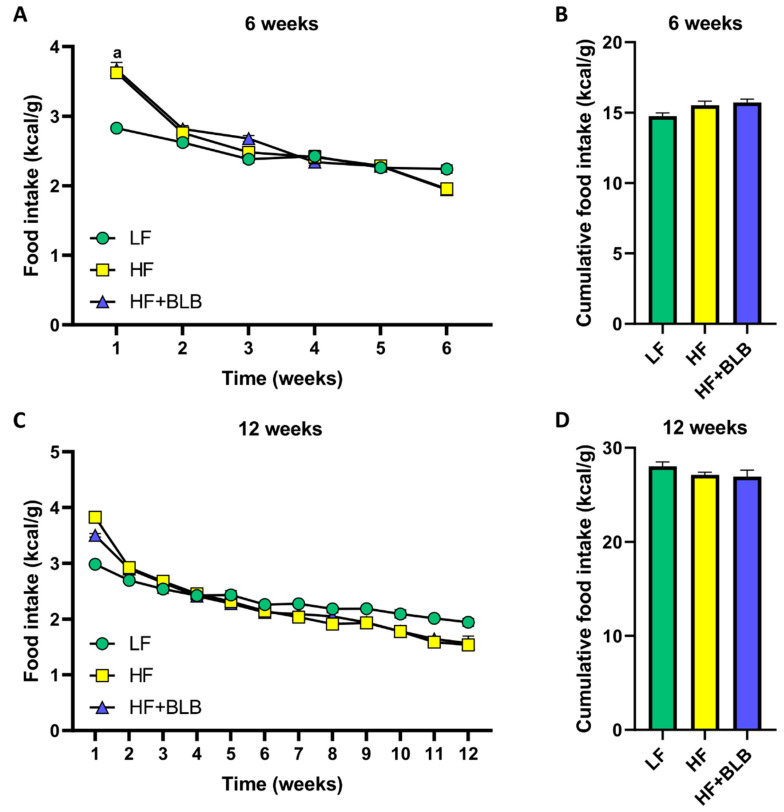
The food intake. The mice received a low-fat diet (LF, 10% of energy from fat, circle), a high-fat diet (HF, 46% of energy from fat, square) or a bilberry-supplemented high-fat diet (HF+BLB, 20% air-dried bilberry powder, triangle) for six (**A**,**B**) or twelve (**C**,**D**) weeks. The weekly food intake was analyzed with repeated measures, such as a two-way (mixed model) ANOVA and a Bonferroni multiple comparisons test (**A**,**C**). The cumulative food intake was analyzed with a one-way ANOVA and a Bonferroni multiple comparisons test (**B**,**D**). The statistically significant difference between the HF and LF groups in the first week is marked as a = *p* < 0.05. The results are expressed as energy unit per body weight (kcal/g); values represent mean ± SEM, and n = 6 for the statistical analysis since the mice were housed as two in a cage; n = 12 mice in each group.

There were no disparities between food intake measured as energy unit per body weight after the first week (Figure 2A,C). The cumulative food intake showed no differences in either six-week or twelve-week treatments (Figure 2B,D).

### 2.2. Epididymal Fat, Liver, and Lipids

Epididymal fat pads represent the metabolically active, harmful visceral fat in the mouse [11]. Another indirect measurement of lipid accumulation is the weight of the liver, which increases when excess fat is gathered in the liver. The HF diet increased the average amount of epididymal fat and the average liver weight, and the bilberry supplementation was unable to prevent these effects in either timepoint. (Table 1).

**Figure 3:**
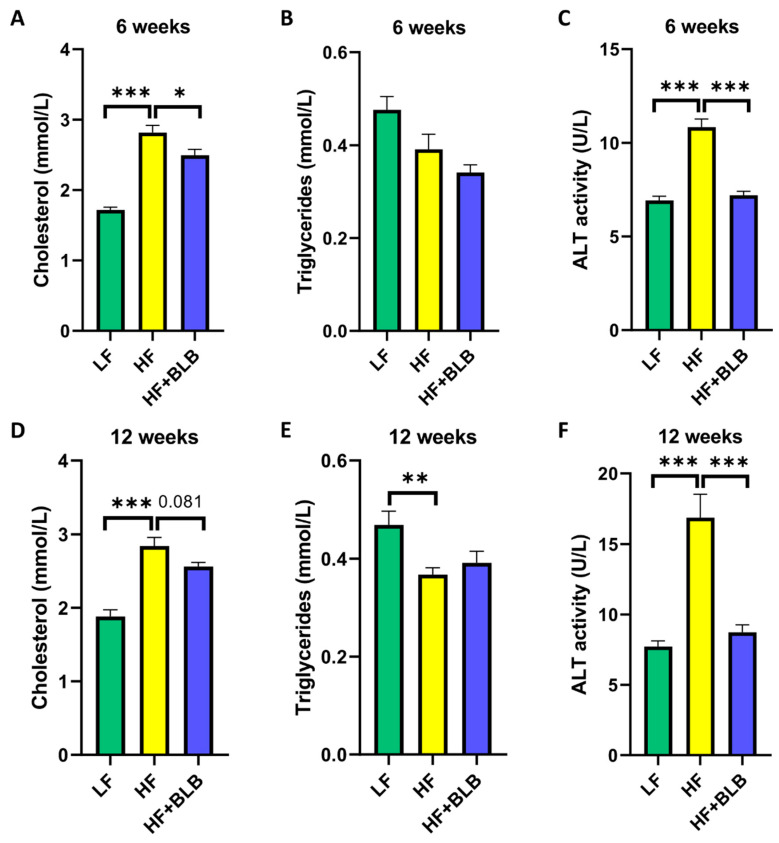
Fasting cholesterol, triglyceride levels, and alanine aminotransferase (ALT) activity. The mice received a low-fat diet (LF, 10% of energy from fat), a high-fat diet (HF, 46% of energy from fat) or a bilberry-supplemented high-fat diet (HF+BLB, 20% air-dried bilberry powder) for six (**A**–**C**) or twelve (**D**–**F**) weeks. The fasting cholesterol levels (**A**,**D**), triglyceride levels (**B**,**E**), and ALT activity (**C**,**F**) were measured from serum and analyzed with a one-way ANOVA and a Bonferroni multiple comparisons test. The statistically significant differences from the HF group are marked with * *p* < 0.05, ** *p* < 0.01, and *** *p* < 0.001. The results are expressed as mmol/L for cholesterol and triglycerides and U/L for ALT activity; values represent mean + SEM; n = 12 mice in each group.

As expected, the HF diet increased serum fasting cholesterol levels after six weeks, being on average 1.72 ± 0.04 mmol/L in the LF group and 2.82 ± 0.10 mmol/L in the HF group (*p* < 0.001). The average cholesterol in the bilberry group was 2.50 ± 0.09 mmol/L, differing significantly from the HF group (*p* < 0.05) and suggesting that the bilberry supplementation partly prevented the cholesterol rise (Figure 3A). After twelve weeks, the average cholesterol in the HF+BLB group (2.56 ± 0.06 mmol/L) continued to be lower than that in the HF group (2.84 ± 0.12 mmol/L), but the difference did not reach statistical significance (*p* = 0.081). (Figure 3D). In triglyceride levels, there was no statistically significant difference between the HF and HF+BLB groups in either timepoint. (Figure 3B,E).

Circulating alanine aminotransferase (ALT) is widely used as a marker of liver injury and inflammation. After six weeks, the ALT activity in the LF group was 6.93 ± 0.22 U/L and that in the HF group 10.8 ± 0.44 U/L (*p* < 0.001). The ALT activity was 7.21 ± 0.21 U/L in the HF+BLB group, indicating that the bilberry supplementation prevented the ALT rise in a statistically significant manner (*p* < 0.001) (Figure 3C). At week twelve, the difference between the LF and HF groups was further increased (*p* < 0.001), the values being 7.73 ± 0.39 U/L for LF and 16.9 ± 1.67 U/L for HF. In the bilberry group, the ALT activity was 8.72 ± 0.54 U/L, which is statistically significantly different from the HF group (*p* < 0.001) (Figure 3F) but not from the LF group, indicating that the bilberry supplementation nearly totally prevented the HF-diet-induced liver injury.

### 2.3. Glucose Metabolism

**Figure 4:**
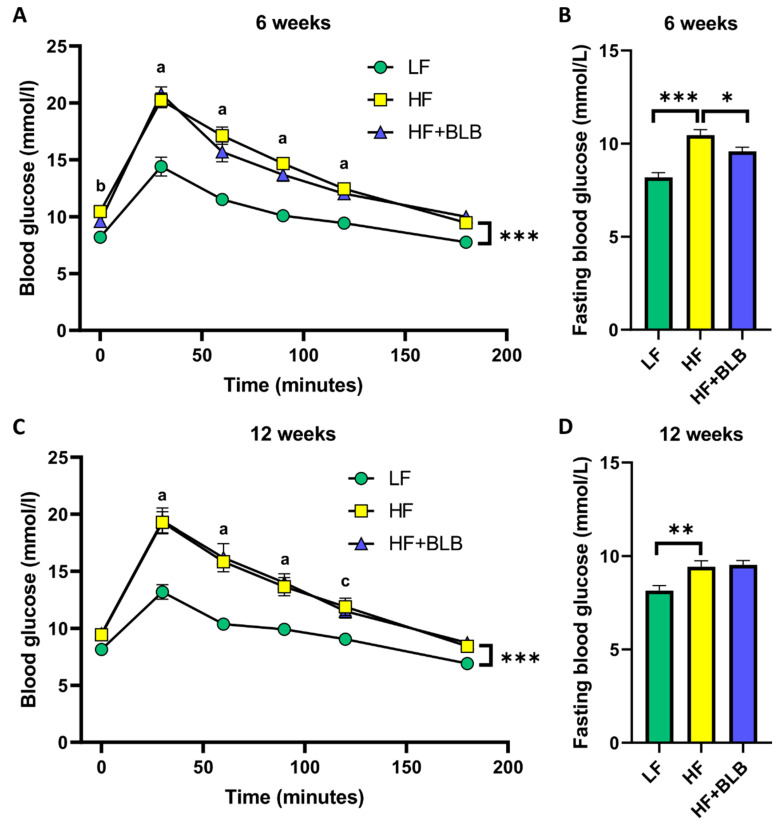
Intraperitoneal glucose tolerance test (IPGTT) and fasting blood glucose levels. The mice received a low-fat diet (LF, 10% of energy from fat, circle), a high-fat diet (HF, 46% of energy from fat, square) or a bilberry-supplemented high-fat diet (HF+BLB, 20% air-dried bilberry powder, triangle) for six (**A**,**B**) or twelve (**C**,**D**) weeks. The IPGTT was carried out a week before the end of each experiment, i.e., at weeks five (**A**) and eleven (**C**). The IPGTT data (**A**,**C**) was analyzed with a two-way (mixed model) ANOVA and a Bonferroni multiple comparisons test. The statistically significant difference between the HF and LF groups in each week is marked as a = *p* < 0.001, b = *p* < 0.01, and c = *p* < 0.05. The fasting glucose data (**B**,**D**) was analyzed with a one-way ANOVA and a Bonferroni multiple comparisons test. The statistically significant differences from the HF group are marked with * *p* < 0.05, ** *p* < 0.01, and *** *p* < 0.001. The results are expressed as mmol/L; values represent mean ± SEM; n = 12 mice in each group.

An intraperitoneal glucose tolerance test was carried out a week before the end of each treatment, i.e., at weeks five and eleven. The mean fasting glucose level at week five in the LF group was 8.19 ± 0.238 mmol/L. In the HF group, it was significantly (*p* < 0.001) elevated to 10.5 ± 0.282 mmol/L, and bilberry prevented the glucose increase in a statistically significant manner (9.58 ± 0.214 mmol/L, *p* < 0.05) at week five (Figure 4B). At week eleven, the average glucose level in the LF group was 8.15 ± 0.264 mmol/L and in the HF group 9.43 ± 0.310 mmol/L, the difference between the groups being statistically significant (*p* < 0.01). In the bilberry group, the average fasting glucose at week eleven was 9.53 ± 0.222 mmol/L, which does not differ statistically significantly from the HF group (Figure 4D).

In the IPGTT test, the highest blood glucose value, which was measured 30 min after the intraperitoneal glucose bolus, was significantly higher in the HF group than in the LF group (*p* < 0.001) (Figure 4A,C). There was no difference between the HF and HF+BLB groups. When comparing area-under-the-curve values between the groups, the difference between the LF and HF groups was statistically significant at week five (*p* < 0.01) and week eleven (*p* < 0.001), but the differences between the HF and HF+BLB groups were statistically insignificant in both timepoints.

**Figure 5:**
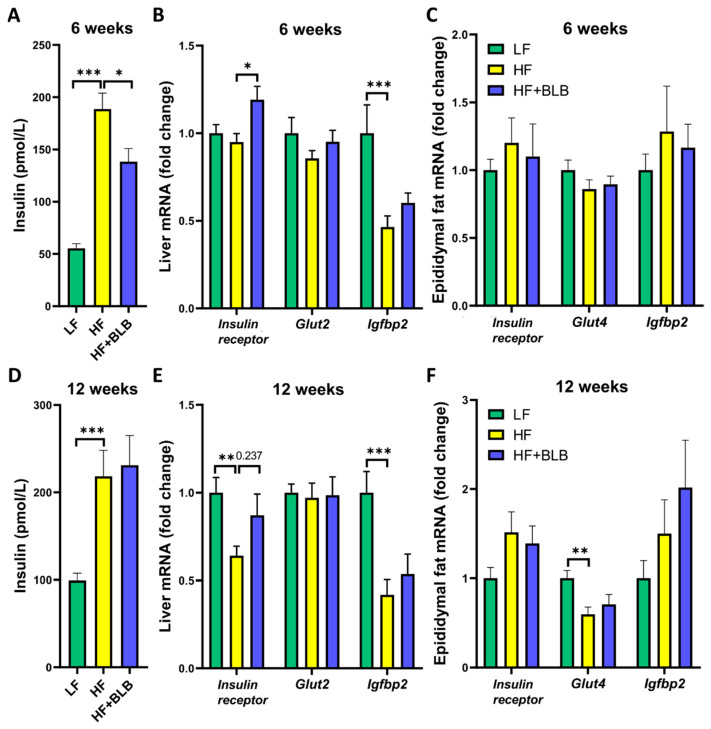
Insulin concentration, insulin receptor, glucose transporter, and insulin-like growth factor binding protein expression. The mice received a low-fat diet (LF, 10% of energy from fat), a high-fat diet (HF, 46% of energy from fat) or a bilberry-supplemented high-fat diet (HF+BLB, 20% air-dried bilberry powder) for six (**A**–**C**) or twelve (**D**–**F**) weeks. The serum insulin was measured by immunoassay at weeks six (**A**) and twelve (**D**). The expression of liver insulin receptor, glucose transporter 2 (*Glut2*), and insulin-like growth factor binding protein 2 (*Igfbp2*) genes at weeks six (**B**) and twelve (**E**) and epididymal fat insulin receptor, glucose transporter 4 (*Glut4*), and insulin-like growth factor binding protein 2 (*Igfbp2*) genes at weeks six (**C**) and twelve (**F**) was measured with RT–PCR and normalized against the housekeeping gene glyceraldehyde 3-phosphate dehydrogenase (*Gapdh*). The expression levels were then compared to the mean expression level of the LF group, which was set to 1. The data was analyzed with a one-way ANOVA and a Bonferroni multiple comparisons test. The statistically significant differences from the HF group are marked with * *p* < 0.05, ** *p* < 0.01, and *** *p* < 0.001. The results are expressed as pmol/L for insulin and as fold change for gene expression. Values represent mean + SEM; n = 12 mice in each group.

In accordance with the results on fasting glucose levels, bilberry also prevented an insulin rise at week six (*p* < 0.05, Figure 5A) and increased insulin receptor gene expression exceeding the LF level in the hepatic (*p* < 0.05, Figure 5B) but not in the visceral adipose tissue (Figure 5C). This suggests that bilberry prevents the development of insulin resistance in the liver by increasing insulin receptor expression. However, the bilberry effect on insulin vanished, and the difference between the HF and HF+BLB groups did not reach significance at week twelve (Figure 5D). The hepatic expression of glucose transporter 2 (*Glut2*) and the adipose tissue expression of glucose transporter 4 (*Glut4*) were unaffected by the bilberry supplementation. Bilberry had no effect on the expression of insulin-like growth factor binding protein 2 (*Igfbp2*) in either of the two tissues (Figure 5B,C,E,F).

### 2.4. Adipokines and Other Inflammatory Factors

Liver and fat tissue produce adipokines—factors involved in the regulation of energy metabolism and appetite. The average level of circulating leptin and the expression of the leptin gene in epididymal fat were elevated in the HF group compared to the LF group at both timepoints (Table 2 and Table 3). The HF diet decreased the expression of the resistin gene in the epididymal fat at week twelve, but the effect on the circulating resistin levels was not statistically significant (Table 2 and Table 3). Despite not affecting the circulating levels of adipokines leptin and adiponectin, bilberry prevented the decrease of circulating adipsin concentration, and the difference was statistically significant at week six (Table 2).

**Figure 6:**
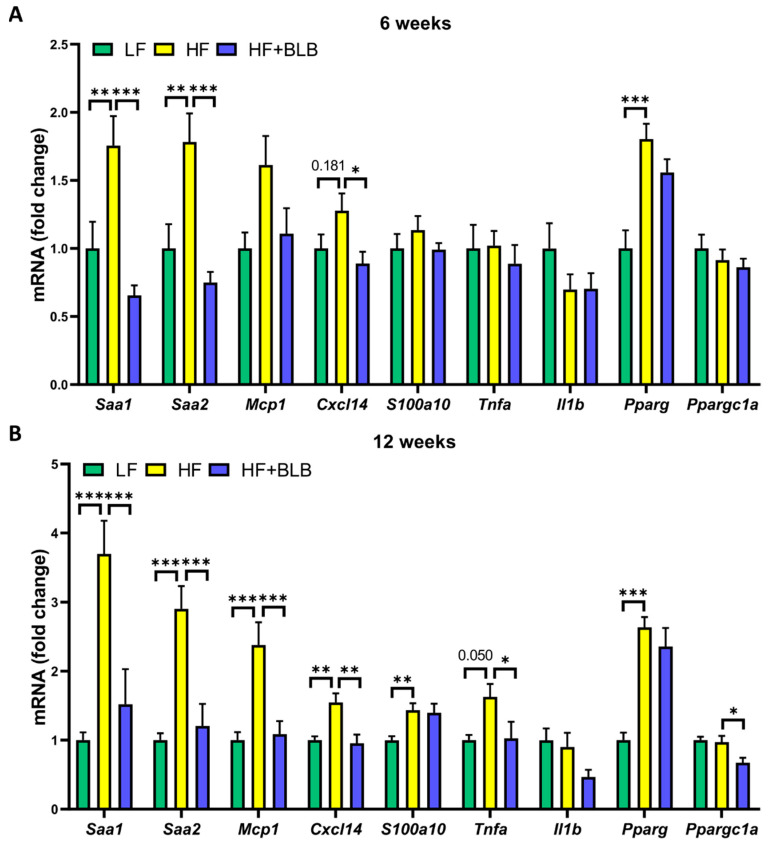
Gene expression in the hepatic tissue. The mice received a low-fat diet (LF, 10% energy from fat), a high-fat diet (HF, 46% of energy from fat) or a bilberry-supplemented high-fat diet (HF+BLB, 20% air-dried bilberry powder) for six (**A**) or twelve (**B**) weeks. The gene expression was measured with RT–PCR and normalized against the housekeeping gene glyceraldehyde 3-phosphate dehydrogenase (*Gapdh*). The expression levels were then compared to the mean expression level in the LF group, which was set to 1. The results are expressed as a fold change and analyzed with a one-way ANOVA and a Bonferroni multiple comparisons test. The statistically significant differences from the HF group are marked with * *p* < 0.05, ** *p* < 0.01, and *** *p* < 0.001. Values represent mean + SEM; n = 12 mice in each group. *Saa1* = Serum amyloid A1, *Saa2* = Serum amyloid A2, *Mcp1* = Monocyte chemoattractant protein 1, *Ccxl14* = Chemokine (C-X-C motif) ligand 14, *S100a10* = S100 calcium-binding protein A10, *Tnfa* = Tumor necrosis factor α, *Il1b* = Interleukin-1β, *Pparg* = Peroxisome proliferator-activated receptor γ, *Ppargc1a* = Peroxisome proliferator-activated receptor γ coactivator 1 α.

**Figure 7:**
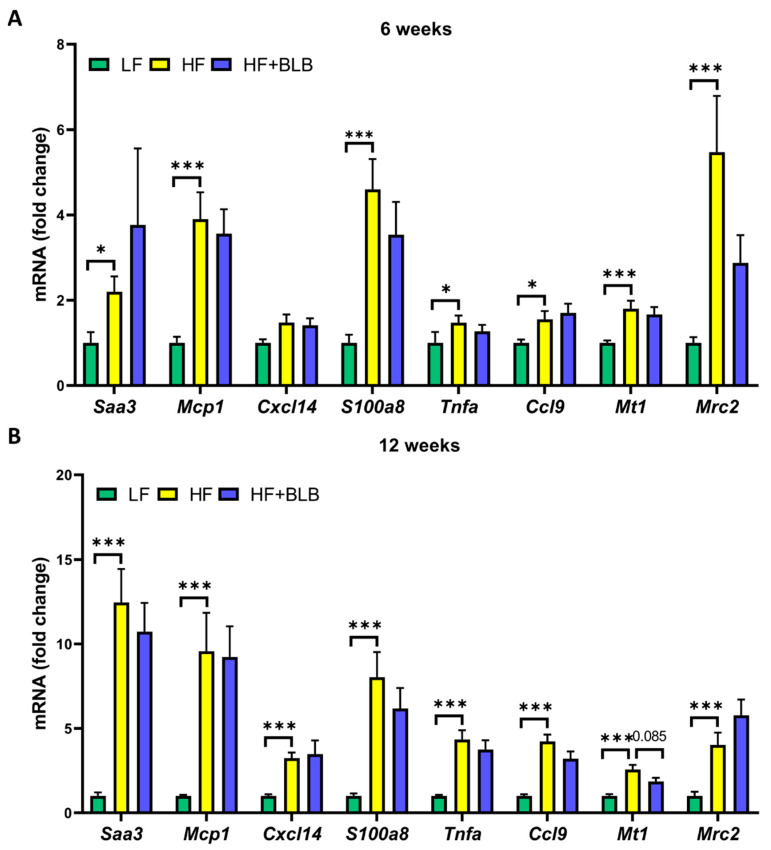
Gene expression in the epididymal fat tissue. The mice received a low-fat diet (LF, 10% energy from fat), a high-fat diet (HF, 46% of energy from fat) or a bilberry-supplemented high-fat diet (HF+BLB, 20% air-dried bilberry powder) for six (**A**) or twelve (**B**) weeks. The gene expression was measured with RT–PCR and normalized against the housekeeping gene glyceraldehyde 3-phosphate dehydrogenase (*Gapdh*). The expression levels were then compared to the mean expression level in the LF group, which was set as 1. The results are expressed as a fold change and analyzed with a one-way ANOVA and a Bonferroni multiple comparisons test. The statistically significant differences from the HF group are marked with * *p* < 0.05 and *** *p* < 0.001. Values represent mean + SEM, n = 12 mice in each group. *Saa3* = serum amyloid A3, *Mcp1* = monocyte chemoattractant protein 1, *Cxcl14* = chemokine (C-X-C motif) ligand 14, *S100a8* = S100 calcium-binding protein A8, *Tnfa* = tumor necrosis factor α, *Ccl9* = chemokine (C-C motif) ligand 9, *Mt1* = metallothionein 1, *Mrc2* = mannose-receptor C type 2.

In obesity, liver and fat tissue produce multiple inflammation-related factors in addition to the classical adipokines. At week six, the HF diet elevated the expression of serum amyloid A1 and A2 (*Saa1* and *Saa2*) and peroxisome proliferator-activated receptor γ (*Pparg*) in the hepatic tissue in a statistically significant manner (Figure 6). The bilberry supplementation significantly prevented the increase in the expression of *Saa1* and *Saa2*. In addition to these findings, at week twelve, the bilberry supplementation prevented the increase in the expression of monocyte chemoattractant protein 1 (*Mcp1*), tumor necrosis factor α (*Tnfa*), and chemokine (C-X-C motif) ligand 14 (*Cxcl14*). The inhibitory effects of bilberry on *Saa1* and *Saa2* expression in the liver were also reflected as lower circulating SAA concentrations in the HF+BLB group compared to the HF group (Table 2). In the adipose tissue, the HF diet had a clear influence on the factors studied, but there were no statistically significant differences between the HF and HF+BLB groups at either timepoint (Figure 7).

## 3. Discussion

**Figure 8:**
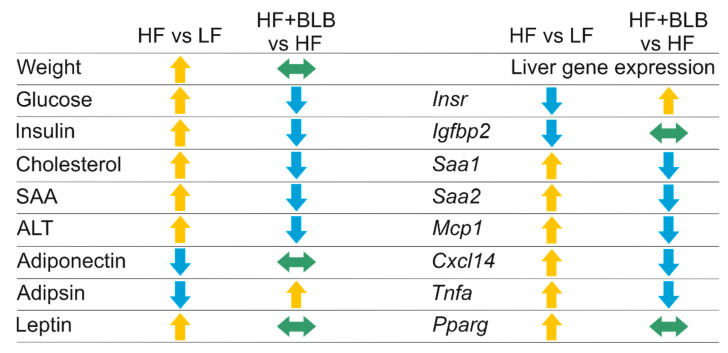
A summary of the effects of bilberry. In the column HF vs. LF, arrows that point upwards and downwards indicate higher or lower levels in the HF group than in the LF group, respectively (i.e., increase or decrease by the HF diet). In the column HF+BLB vs. HF, arrows that point upwards and downwards indicate higher and lower levels in the HF+BLB group than in the HF group, respectively (i.e., the prevention of the HF-diet effect by the bilberry supplementation). Arrows pointing sideways indicate no change in the parameter or gene expression. LF = low-fat diet, HF = high-fat diet, HF+BLB = bilberry-supplemented high-fat diet, SAA = serum amyloid A, ALT = alanine aminotransferase, *Insr* = insulin receptor, *Igfbp2* = insulin-like growth factor binding protein 2, *Saa1* = serum amyloid A1, *Saa2* = serum amyloid A2, *Mcp1* = monocyte chemoattractant protein 1, *Cxcl14* = chemokine (C-X-C motif) ligand 14, *Tnfa* = tumor necrosis factor α, *Pparg* = peroxisome proliferator-activated receptor γ coactivator 1 α.

The effects of dried bilberry (*Vaccinium myrtillus* L.) on glucose and lipid metabolism and low-grade inflammation were studied in a high-fat-diet-induced model of obesity in mice. The bilberry supplementation was found to prevent the rise of circulating cholesterol, glucose, ALT, and SAA levels, as well as the expression of several inflammation-related genes in the liver (Figure 8). These data indicate that bilberry was able to partly resist the development of hypercholesterolemia, hyperglycemia, and systemic and hepatic inflammation induced by the HF diet in the mouse model. The HF and HF+BLB diets were designed to have matched amounts of energy and major nutritional ingredients per mass unit. As there were no differences in the energy intake or weight gain between the HF and HF+BLB groups, the observed differences in the metabolic and inflammatory parameters are most likely caused by the bilberry supplementation *per se* and not by less severe obesity, for instance. Thus, bilberry seems to be capable of shifting the metabolic balance towards healthier obesity, which is known to reduce the risk of obesity-associated diseases such as type II diabetes and cardiovascular diseases [1].

In the present study, bilberry supplementation prevented the HF-diet-induced increase in the circulating cholesterol levels by about 30% (*p* < 0.05 at six weeks and *p* = 0.081 at twelve weeks). In corresponding experimental models, the effect of the well-known cholesterol-lowering drug simvastatin was at about a similar level [12,13]. These data suggest that the detected effect of bilberry supplementation on serum cholesterol levels is likely to be of clinical relevance, and further human studies are therefore encouraged. Circulating triglyceride concentrations were also measured. Triglyceride levels typically increase in humans as a consequence of a high fat content in the diet [14]. However, in mice, a HF diet normally either decreases circulating triglycerides or has no effect on them [15,16,17]. The former was also seen in the present study, but the bilberry supplementation had no consistent effect on triglyceride levels.

Bilberry supplementation also had a positive impact on fasting blood glucose and insulin levels, as well as on IPGTT at week six supporting the findings in previous studies [18,19,20]. These parameters are regarded as signs of developing insulin resistance and type II diabetes. In the present study, bilberry supplementation prevented the HF-diet-induced increase in the fasting blood glucose by about 40% at week six. The effect was about at the same level as that reported by the widely used glucose-lowering drug, metformin [21,22]. Our findings were accompanied by the increased expression of insulin receptor in the liver. This may explain the observed reduced glucose and insulin concentrations in the HF+BLB group, because the level of insulin receptor expression is a significant determinant of insulin effects [2]. Accordingly, insulin-resistant hepatocytes have been reported to express insulin receptors at reduced levels [23,24]. However, as bilberry caused no significant differences in insulin receptor expression in the visceral adipose tissue, its actions seem to be tissue-specific, possibly for pharmacokinetic reasons (see below). The expression of the insulin-insensitive glucose transporter *Glut2* [2] in the liver, insulin-sensitive glucose transporter *Glut4* [2] in the visceral adipose tissue or *Igfbp2* in either of those tissues did not differ between the HF and HF+BLB groups. Thus, bilberry supplementation could retard and partially prevent the development of insulin resistance.

In the present study, the effect of bilberry supplementation on circulating cholesterol, glucose, and insulin levels was more prominent at week six than week twelve. Thus, the effect of bilberry on these parameters seemed to decline with time. In the clinical context, it is a common phenomenon that type II diabetes or hypercholesterolemia progresses despite adequate medication, particularly without concomitant appropriate lifestyle changes. Therefore, there is often a need to reinforce the medication during the course of the disease by increasing doses and/or by adding additional drugs to the treatment. As discussed above, the efficacy of bilberry supplementation at week six in the present study was close to that of simvastatin and metformin in similar models [12,13,21,22].

In addition to the beneficial effects on cholesterol and glucose metabolism, bilberry supplementation nearly totally prevented the HF-diet-induced increase in circulating ALT levels, a general marker of hepatocyte injury and liver inflammation. In addition, bilberry supplementation prevented the HF-diet-induced increase in the expression of inflammatory factors in the liver. Obesity is associated with NAFLD, wherein metabolic changes lead to liver inflammation and further to liver cirrhosis in severe cases [4]. Increased dietary energy content and insulin resistance in adipose tissue cause the excessive hepatic intake of lipids that are partly metabolized to toxic metabolites [25,26]. The lipotoxicity leads to mitochondrional dysfunction, oxidative stress, and hepatocyte injury. This activates liver resident macrophages (Kupffer cells), infiltrated leukocytes, and injured hepatocytes to produce cytokines and chemokines that maintain and accelerate the inflammatory process [4,26].

In the present study, the hepatic expression of inflammatory factors *Tnfa, Mcp1*, *Cxcl14*, *Saa1*, and *Saa2* was significantly lower in the HF+BLB group compared to the HF group. The result indicates that bilberry decelerates the development of liver inflammation. One possible explanation for this effect may be the reduced number of inflammatory cells recruited to the hepatic tissue, as the bilberry supplementation prevented the HF-diet-induced increase in the expression of chemokines, particularly *Cxcl14* and *Mcp1*. MCP1 is the main promoter of macrophage and monocyte infiltration to the liver [4,6]. CXCL14 attracts macrophages, immature dendritic cells, natural killer cells, and B cells [27,28] but also promotes M2-type macrophage polarization [29,30].

SAAs are a group of proteins produced in the liver (SAA1 and SAA2) and, in rodents, also in the adipose tissue (SAA3). SAAs are inflammatory acute phase proteins and are elevated in chronic inflammation as well. They have also been linked to the development of atherosclerosis, but the underlining mechanisms remain poorly understood [31]. In our study, bilberry supplementation clearly prevented the rise of circulating SAA and the expression of *Saa1* and *Saa2* in the liver, underlining the anti-inflammatory action of bilberry.

Visceral adipose tissue is metabolically highly active, and its accumulation is related to deleterious conditions, such as systemic inflammation and insulin resistance [1]. A generally used equivalent of the visceral adipose tissue in mouse models are the epididymal fat pads [3]. In the present study, the amount of epididymal fat was significantly higher in the HF group than in the LF group, but there was no difference between the HF and HF+BLB groups. This result is supported by earlier observations in mouse and rat HF-diet models supplemented with bilberry [18,19,20,32,33]. In the visceral adipose tissue, bilberry’s effects on inflammatory factors or adipokine expression were sparse. Since adipose tissue is the major source of the studied adipokines [5,34], it is not surprising that bilberry supplementation did not cause major changes in their circulating levels. Still, the circulating adipsin levels were higher in the HF+BLB group than in the HF group. Interestingly, adipsin has recently been shown to alleviate hyperglycemia and to protect beta cells in a mouse model of type II diabetes [35]. Despite the mechanism of adipsin regulation by bilberry supplementation remaining unclear, this finding is in accordance with our general hypothesis of the metabolically beneficial actions of bilberry.

Bilberry is remarkably rich in polyphenols, particularly anthocyanins [36,37]. Anthocyanins usually exist in plants as glycosides and their noncarbohydrate moieties, anthocyanidins [9]. Anthocyanins are red-, blue-, purple-, and black-colored pigments formed in the plant during the growing period and regulated by genetic and environmental factors [8,38]. Berry anthocyanins and flavonoids exhibit antioxidant and anti-inflammatory properties [39,40]. The anthocyanin profile of bilberry has been reported to include fifteen groups of anthocyanin glycosides, the main groups being delphinidin, cyanidin, petunidin, malvidin, and peonidin [36,38,41,42,43,44]. Bilberry also contains flavonoids kaempferol and quercetin, as well as condensed tannins, namely proanthocyanidins at lower concentrations [42,45,46].

Anthocyanins have been investigated in vitro and in vivo. Delphinidin was reported to inhibit lipid accumulation and cyanidin reactive oxygen species production in HepG2 hepatocytes [47,48]. Malvidin was found to reduce the development of liver fibrosis by inducing hepatic stellate cell apoptosis and to inhibit TNFα and interleukin (IL)-1β secretion from activated macrophages [49,50]. A combination of malvidin and peonidin was reported to downregulate the expression of inflammation-related genes in adipocytes exposed to lipopolysaccharide [51]. A bilberry–blackcurrant-derived anthocyanin preparation alleviated oxidative stress and inflammation in human diabetic endothelial cells (D-HAEC) [52]. In animal studies, cyanidin was found to suppress body fat accumulation induced by a HF diet and to attenuate oxidative stress in the livers of diabetic mice [48,53], while a combination of cyanidin and delphinidin alleviated the obesity, dyslipidemia, and insulin resistance caused by HF diet [54]. An anthocyanin preparation from bilberries was reported to improve insulin sensitivity in type II diabetic mice [55]. These direct effects of bilberry anthocyanins may well contribute to the effects observed in the current study. In addition, bilberry polyphenols may alter the gut microbiome and thus secondarily influence the response to obesity-inducing diets [32,56,57,58]. Further studies are needed to understand the effective constituents of bilberry and their detailed mechanisms of action.

In the present study, the lack of bilberry effects on adipose tissue was remarkable compared to the effects on the liver. The intestine drains via the portal vein to the liver bringing the nutrients and compounds absorbed in the intestine, and many of them are significantly metabolized in the liver. This suggests that the concentration of many of the bilberry-specific molecules is higher in the hepatic tissue than in other tissues in the body. In our study, the expression of *Cxcl14*, *Tnfa*, *Mcp1,* and *Saas* was affected by bilberry supplementation in the liver but not in the epididymal fat. This finding is supported by the results of Sakakibara et al. [59]. They fed mice bilberry extract mixed with a normal rodent diet for two weeks and detected bilberry anthocyanins in the blood and in the liver but not in the adipose tissue. The low concentration or absence of anthocyanins in adipose and muscle tissues could also explain the partial effect of bilberry on the glucose metabolism found in the present study, as discussed above.

To our knowledge, this is the first study wherein the effects of bilberry supplementation on the expression of adipokines and inflammatory factors in the adipose and hepatic tissues have been studied to this extent. Thus, our results extend the previous knowledge of the effects of bilberry by providing new information on the inflammatory changes in the liver. The experiments of the present study are best applied to developing obesity and may not fully apply if obesity is clearly already established. Further studies are needed to better evaluate bilberry efficacy in the latter situation. Regarding clinical importance, bilberry is considered safe [10]. In a systematic review of clinical studies, the daily intake of anthocyanins (the main group of phenolic compounds in bilberry) went up to 640 mg/day, and no adverse effects were reported [60]. There are few clinical studies on the effects of bilberry supplementation in individuals with type II diabetes, obesity, or metabolic syndrome, their results supporting the findings of the present study [61,62]. Based on the current results and the previous evidence, bilberries can be recommended as a part of healthy diet, despite further studies being needed to confirm the current findings in humans and to investigate the exact mechanisms and active components of bilberry.

In conclusion, bilberry supplementation retarded/prevented the rise of circulating cholesterol, glucose, ALT, SAA, and the increase in the expression of many inflammation-related genes in the liver induced by a HF diet in a mouse model of obesity. Based on these results, dietary bilberry has a beneficial effect on the development of hypercholesterolemia and liver inflammation. In addition, bilberry supplementation had a partial impact on the glucose metabolism disturbed by a high dietary fat content. The polyphenols of the bilberry powder are a plausible explanation for the anti-inflammatory effects detected in the present study.

## 4. Materials and Methods

### 4.1. Animals and Study Design

At eight weeks of age male C57BL/6N mice (Scanbur Research A/S, Karlslunde, Denmark) were divided into groups of twelve mice (2 mice/cage) and maintained for six or twelve weeks on a low-fat diet (LF diet, 10% of energy from fat), a high-fat diet (HF diet, 46% of energy from fat) or an air-dried-bilberry-powder-supplemented (20% *w*/*w*) high-fat diet (HF+BLB diet). The bilberry powder was produced from Finnish bilberries, and 900 g of fresh berries was used to produce on average 100 g of powder (Kiantama Oy, Suomussalmi, Finland).

The nutrient composition of the bilberry powder was taken into account when custom-made diets were designed in collaboration with the manufacturer (Research Diets, Inc., New Brunswick, NJ, USA). All diets were matched for their fiber, protein, and other ingredients, as well as carbohydrates and fats for HF diets (Table 4).

Mice body weights and food consumption were followed with weekly measurements, and at the end of the study, serum and tissue samples were collected for further analyses. Mice were housed in the animal facility of Tampere University under standard conditions (12 h light/dark cycle, temperature 22 ± 1 °C, humidity 50–60%) with food and water provided *ad libitum*. The study was approved by the National Animal Experimental Board (ESAVI/984/04.10.07/2018), and the experiments were carried out in accordance with the EU legislation for the protection of animals used for scientific purposes (Directive 2010/63/EU).

### 4.2. Intraperitoneal Glucose Tolerance Test

An intraperitoneal glucose tolerance test (IPGTT) was performed for both six- and twelve-week study groups one week before the end of the experiment, i.e., at weeks five and eleven, respectively. Mice were on morning fast for six hours, and thereafter their baseline blood glucose was measured from the tip of the tail with a Contour Next One glucometer (Oy Diabet Ab, Lemu, Finland). Sterile filtered glucose in PBS (2 g/kg, Sigma-Aldrich, St. Louis, MO, USA) was injected intraperitoneally into the mice, and blood glucose levels were measured at 30, 60, 90, 120, and 180 min following the injection.

### 4.3. Blood Samples and Analyses

At the end of the study, mice were fasted for six hours (morning fast), and blood was collected under anesthesia by cardiac puncture; thereafter, the mice were euthanized by cervical dislocation. Blood samples were centrifuged for 15 min at 1500× *g* after 30 min incubation at room temperature, and the serum was immediately stored at −80 °C. Serum total cholesterol and triglyceride levels and alanine aminotransferase (ALT) activity were measured by fluorometric assays (Abcam, Cambridge, UK). Enzyme-linked immunoassays were used to measure the concentrations of serum amyloid A, leptin, resistin, adipsin and adiponectin (R & D Systems Europe Ltd., Abingdon, UK), and insulin (Mercodia Ltd., Uppsala, Sweden) in the serum samples. Detection limits were 62.5 pg/mL for serum amyloid A, 7.8 pg/mL for leptin and resistin, 375 pg/mL for adipsin, 15.6 pg/mL for adiponectin, and 33 pmol/L for insulin.

### 4.4. RNA Extraction

Liver and epididymal fat samples were stored immediately after collection in RNA Later^®^ (Ambion, Thermo Fisher Scientific, Waltham, MA, USA). For RNA extraction, liver tissue (25–30 mg) was cut into smaller pieces and homogenized with Qiashredder (Qiagen Inc., Hilden, Germany). RNA was extracted with an RNeasy Mini Kit (Qiagen) with on-column DNase digestion (Qiagen). Epididymal fat tissue (125–135 mg) was cut into smaller pieces and homogenized in QIAzol lysis reagent, and the RNA was extracted with RNeasy Lipid Tissue kit with on-column DNase digestion (Qiagen).

### 4.5. Reverse Transcription–Polymerase Chain Reaction (RT–PCR)

RNA was transcribed to cDNA with Maxima First Strand cDNA Synthesis Kit (Thermo Fisher Scientific) and diluted 1:5 with RNase-free water. Quantitative PCR was performed using TaqMan Universal Master Mix and an ABI Prism 7500 sequence detection system (Applied Biosystems, Foster City, CA, USA). The PCR cycling parameters were incubation at 50 °C for 2 min, incubation at 95 °C for 10 min, and thereafter 40 cycles of denaturation at 95 °C for 15 s and annealing and extension at 60 °C for 1 min. Primers and probe for the housekeeping gene glyceraldehyde 3-phosphate dehydrogenase (*Gapdh*) were GCATGGCCTTCCGTGTTC (forward, 300 nM), GATGTCATCATACTTGGCAGGTTT (reverse, 300 nM), and TCGTGGATCTGACGTGCCGCC (probe, 150 nM); those for tumor necrosis factor α (*Tnfa*) were GACCCTCACACTCAGATCATCTTCT (forward, 900 nM), CCTCCACTTGGTGGTTTGCT (reverse, 300 nM), and AAAATTCGAGTGACAAGCCTGTAGCCCA (probe, 200 nM). The sequences and concentrations were optimized according to the manufacturer’s guidelines in TaqMan Universal PCR Master Mix Protocol part number 4304449 revision C (Applied Biosystems). In addition, the TaqMan Gene Expression assays (Thermo Fisher Scientific) listed on Table 5 were used, and the expression levels were calculated using the 2(−ΔΔCT) method. When calculating results, all mRNA levels were first normalized against *Gapdh* mRNA levels.

### 4.6. Statistics

Results are expressed as mean ± standard error of mean (SEM). The distribution of each data group was analyzed with a D’Agostino and Pearson omnibus normality test, and, when the assumption of normality was not met, the data was transformed to achieve a normal distribution. One-way and two-way analyses of variance (ANOVA) with Bonferroni multiple comparisons test were used in the statistical analysis. When calculating the results of the IPGTT, the area under the curve (AUC) was calculated for each group using the fasting glucose level of the LF group as the zero level. The analyses were conducted and the graphs drawn with GraphPad Prism version 8.3.0 (GraphPad Software Inc., San Diego, CA, USA).

## Figures and Tables

**Table 1:** Epididymal fat and liver weight. The mice received a low-fat diet (LF, 10% of energy from fat), a high-fat diet (HF, 46% of energy from fat) or a bilberry-supplemented high-fat diet (HF+BLB, 20% air-dried bilberry powder) for six or twelve weeks. The weights were analyzed with a one-way ANOVA and a Bonferroni multiple comparisons test. The results are expressed as grams; values represent mean ± SEM; n = 12 mice in each group; ns = not significant (*p* > 0.05).

Tissue	Low-Fat Diet (LF)	High-Fat Diet (HF)	Bilberry Supplemented High-Fat Diet (HF+BLB)	*p*-Value between LF and HF	*p*-Value between HF and HF+BLB
Week 6					
Epididymal fat (g)	0.84 ± 0.04	2.01 ± 0.09	1.99 ± 0.08	<0.001	ns
Liver (g)	1.01 ± 0.03	1.29 ± 0.05	1.18 ± 0.04	<0.001	ns
Week 12					
Epididymal fat (g)	1.16 ± 0.09	2.82 ± 0.11	2.81 ± 0.12	<0.001	ns
Liver (g)	1.20 ± 0.05	1.45 ± 0.11	1.37 ± 0.05	0.0780	ns

**Table 2:** The levels of circulating adipokines and serum amyloid A (SAA). The mice received a low-fat diet (LF, 10% energy from fat), a high-fat diet (HF, 46% of energy from fat) or a bilberry-supplemented high-fat diet (HF+BLB, 20% air-dried bilberry powder) for six or twelve weeks. The results are analyzed with a one-way ANOVA and a Bonferroni multiple comparisons test and expressed as mg/L for adiponectin and adipsin and μg/L for leptin, resistin, and SAA. Values represent mean ± SEM; n = 12 mice in each group; ns = not significant (*p* > 0.05).

Protein	Low-Fat Diet (LF)	High-Fat Diet (HF)	Bilberry Supplemented High-Fat Diet (HF+BLB)	*p*-Value between LF and HF	*p*-Value between HF and HF+BLB
Week 6					
Adiponectin (mg/L)	7.43 ± 0. 20	6.20 ± 0.12	6.21 ± 0.49	<0.001	ns
Adipsin (mg/L)	11.7 ± 0.38	7.48 ± 0.33	9.36 ± 0.37	<0.001	<0.01
Leptin (μg/L)	3.52 ± 6.88	25.8 ± 2.25	21.8 ± 2.52	<0.001	ns
Resistin (μg/L)	17.8 ± 0.69	16.5 ± 0.58	16.7 ± 0.47	ns	ns
SAA (μg/L)	219 ± 21.3	388 ± 33.2	201 ± 13.0	<0.001	<0.001
Week 12					
Adiponectin (mg/L)	6.56 ± 0.12	6.35 ± 0.20	6.72 ± 0.25	ns	ns
Adipsin (mg/L)	9.99 ± 0.38	6.90 ± 0.55	7.67 ± 0.38	<0.001	ns
Leptin (μg/L)	6.88 ± 1.09	39.1 ± 4.47	39.1 ± 2.52	<0.001	ns
Resistin (μg/L)	18.5 ± 1.14	17.7 ± 0.63	15.8 ± 0.74	ns	ns
SAA (μg/L)	269 ± 24.4	629 ± 87.2	348 ± 106	<0.01	<0.01

**Table 3:** The expression of adipokines in epididymal fat tissue. The mice received a low-fat diet (LF, 10% energy from fat), a high-fat diet (HF, 46% of energy from fat) or a bilberry-supplemented high-fat diet (HF+BLB, 20% air-dried bilberry powder) for six or twelve weeks. The gene expression was measured with RT–PCR and normalized against the housekeeping gene glyceraldehyde 3-phosphate dehydrogenase (*Gapdh*). The expression levels were then compared to the mean expression level of the LF group. The results are expressed as a fold change and analyzed with a one-way ANOVA and a Bonferroni multiple comparisons test. Values represent mean ± SEM; n = 12 mice in each group; ns = not significant (*p* > 0.05).

Gene	Low-Fat Diet (LF)	High-Fat Diet (HF)	Bilberry Supplemented High-Fat Diet (HF+BLB)	*p*-Value between LF and HF	*p*-Value between HF and HF+BLB
Week 6					
Adiponectin	1 ± 0.04	1.00 ± 0.09	1.00 ± 0.078	ns	ns
Adipsin	1 ± 0.07	0.57 ± 0.05	0.73 ± 0.07	< 0.001	ns
Leptin	1 ± 0.09	3.77 ± 0.35	3.52 ± 0.35	< 0.001	ns
Leptin receptor	1 ± 0.10	0.80 ± 0.11	0.93 ± 0.23	ns	ns
Resistin	1 ± 0.05	0.89 ± 0.08	0.86 ± 0.07	ns	ns
Week 12					
Adiponectin	1 ± 0.05	0.85 ± 0.12	0.85 ± 0.07	ns	ns
Adipsin	1 ± 0.08	0.41 ± 0.08	0.46 ± 0.09	< 0.001	ns
Leptin	1 ± 0.15	2.54 ± 0.281	2.80 ± 0.33	< 0.001	ns
Leptin receptor	1 ± 0.18	0.87 ± 0.19	0.93 ± 0.14	ns	ns
Resistin	1 ± 0.06	0.54 ± 0.05	0.47 ± 0.08	< 0.001	ns

**Table 4:** Composition of the experimental diets.

Nutrients	Low-Fat Diet (LF)	High-Fat Diet (HF)	Bilberry Supplemented High-Fat Diet (HF+BLB)
Calculated energy (kcal)			
Protein	716	716	716
Carbohydrate	2840	1422	1422
Fat	405	1823	1823
Total Energy	3961	3961	3961
Calculated Energy per gram diet (kcal/g)	3.72	4.56	4.41
Calculated Energy (kcal%)			
Protein	18	18	18
Carbohydrate	72	36	36
Fat	10	46	46
Total Energy	100	100	100
Bilberry powder (g)	0	0	180 ^1^
Ingredients (g)			
Casein (protein)	200	200	189 (*+ 10 from BLB*), total 199
L-Cystine	3	3	3
Corn Starch	48	68	49 (*+ 23 from BLB*), total 72
Maltodextrin 10	75	100	100
Glucose	28	28	0 (*+ 28 from BLB*), total 28
Fructose	38	38	0 (*+ 38 from BLB*), total 38
Sucrose	107	107	107
Cellulose (insoluble fiber)	54	54	22 (*+ 32 from BLB*), total 54
Inulin (soluble fiber)	12	12	3 (*+ 9 from BLB*), total 12
Soybean Oil	25	25	9 (+ *12 from BLB*), total 21
Lard	20	178	182
Mineral Mix S10026	10	10	10
DiCalcium Phosphate	13	13	13
Calcium Carbonate	6	6	6
Potassium Citrate	17	17	17
Vitamin Mix V10001	10	10	10
Choline Bitartrate	2	2	2

^1^ Nutrient content/100 g bilberry powder: fat 6.7 g, carbohydrates 49.9 g (of which sugars 37.1 g), fiber 23 g (of which soluble 5 g), protein 5.6 g, total anthocyanins 1430 mg.

**Table 5:** TaqMan Gene Expression Assays.

Gene	Abbr.	Assay ID
Adiponectin	*Adipoq*	Mm00456425_m1
Adipsin	*And*	Mm01143935_g1
Chemokine (C-C motif) ligand 9	*Ccl9*	Mm00441260_m1
Chemokine (C-X-C motif) ligand 14	*Cxcl14*	Mm00444699_m1
Glucose transporter 2	*Glut2*	Mm00446229_m1
Glucose transporter 4	*Glut4*	Mm00436615_m1
Insulin like growth factor binding protein 2	*Igfbp2*	Mm00492632_m1
Insulin receptor	*Insr*	Mm01211875_m1
Interleukin 1β	*Il1b*	Mm00434228_m1
Leptin	*Lep*	Mm00434759_m1
Leptin receptor	*Lepr*	Mm00440181_m1
Mannose receptor C type 2	*Mrc2*	Mm00485184_m1
Metallothionein 1	*Mt1*	Mm00496660_g1
Monocyte chemoattractant protein 1	*Mcp1*	Mm00441242_m1
Peroxisome proliferator-activated receptor γ	*Pparg*	Mm01184322_m1
Peroxisome proliferator-activated receptor γ coactivator 1α	*Ppargc1a*	Mm01208835_m1
Resistin	*Retn*	Mm00445641_m1
S100 calcium-binding protein A8	*S100a8*	Mm00496696_g1
S100 calcium-binding protein A10	*S100a10*	Mm00501458_g1
Serum amyloid A1	*Saa1*	Mm00656927_g1
Serum amyloid A2	*Saa2*	Mm04208126_mH
Serum amyloid A3	*Saa3*	Mm00441203_m1

## Data Availability

All of the data are presented in the manuscript.
